# Molecular epidemiology of canine parvovirus type 2 in Italy from 1994 to 2017: recurrence of the CPV-2b variant

**DOI:** 10.1186/s12917-019-2096-1

**Published:** 2019-11-04

**Authors:** Mara Battilani, Francesco Modugno, Francesco Mira, Giuseppa Purpari, Santina Di Bella, Annalisa Guercio, Andrea Balboni

**Affiliations:** 1 Department of Veterinary Medical Sciences, Alma Mater Studiorum – University of Bologna, Via Tolara di Sopra 50, 40064 Ozzano dell’Emilia, BO Italy; 2 Istituto Zooprofilattico Sperimentale della Sicilia “A. Mirri”, Palermo, Italy

**Keywords:** Canine parvovirus, Dog, Epidemiology, Evolution, Phylodynamics

## Abstract

**Background:**

Canine parvovirus type 2 (CPV-2) is the most important enteric virus infecting canids. It is a rapidly evolving virus; after its emergence in the 1970s, new antigenic variants (called CPV-2a, 2b and 2c) emerged and replaced the original antigenic type. The three antigenic variants are globally distributed with different frequencies and levels of genetic variability. This study focused on VP2 gene sequence analysis and the phylodynamics of CPV-2 which were detected in 123 dogs showing clinical signs of gastroenteritis collected in Italy from 1994 to 2017.

**Results:**

For the most part, the sick dogs were young, and a third of them (32.5%) had been vaccinated. No statistical association was found between the CPV-2 antigenic variants, and sex, age, breed and vaccination status. Sequence analysis showed that all three antigenic types circulated in Italy; the CPV-2a type was the prominent genotype, followed by CPV-2c and CPV-2b, with notable differences regarding regional bases and significant fluctuations over time. Nucleotide sequence data showed high genetic heterogeneity with 67 nucleotide sequence types (ntSTs) identified, corresponding to 21 amino acid sequence types (aaSTs). The aaSTs and ntSTs obtained were distributed differently among the three CPV-2 antigenic variants: CPV-2a grouped 12/21 (57.1%) aaSTs and 41/67 (61.2%) ntSTs; CPV-2b grouped 5/21 (23.8%) aaSTs and 6/67 (8.9%) ntSTs, and CPV-2c grouped 4/21 (19.1%) aaSTs and 20/67 (29.9%) ntSTs. Canine parvovirus 2a was characterised by the highest genetic variability while CPV-2c was characterised by notable stability with a predominant amino acid profile during the entire sampling time. Canine parvovirus 2b re-emerged in recent years, showing a new and distinctive amino acid profile of the VP2 protein.

**Conclusions:**

The findings of the present study provided new insights regarding the phylodynamics and evolution of CPV-2 in Italy, pointing out notable differences at the local level in the distribution of the CPV-2 variants and the selection of genetic subtypes. The evolution of CPV-2 has raised questions regarding the efficacy of vaccination; therefore, continuous monitoring regarding the evolution and spread of new CPV-2 variants should be a key aim of ongoing research.

## Background

Canine parvovirus type 2 (CPV-2) is a non-enveloped single-stranded DNA virus belonging to the family *Parvoviridae*, the subfamily *Parvovirinae* and the genus *Protoparvovirus* according to the International Committee on Taxonomy of Viruses (ICTV) [1]. Recently, CPV-2 has been included within the species *Carnivore protoparvovirus* 1 which also includes other closely related autonomous viruses causing a range of serious conditions, especially in young animals: feline panleukopenia virus (FPV, the prototype virus of the former carnivore protoparvovirus), mink enteritis virus (MEV) and raccoon parvovirus (RaPV). These viruses are considered to be host variants of a unique viral species, given the reciprocal high genomic and antigenic relationship [2].

Canine parvovirus type 2 is responsible for acute gastroenteritis in dogs, often fatal in 6–12-week-old puppies. In fact, despite vaccination, it is still widespread in the canine population and, if pups are not vaccinated or when maternal antibodies interfere with their vaccination, they generally become naturally infected [3]. Furthermore, CPV-2 infection has also been reported in vaccinated adult dogs [4].

Although CPV-2 is a DNA virus, its genomic substitution rate is similar to RNA viruses, with a value of approximately 10^− 4^ substitutions per site per year [5]. Consequently, after its emergence in the late 1970s, CPV-2 has been undergoing rapid evolution and, in just a few years, the original antigenic type 2 has been completely replaced by the new antigenic variants called CPV-2a, -2b and -2c, based on key amino acid substitutions in the VP2 protein [6, 7]. These amino acid changes have provided important biological properties and have enabled the CPV-2 variants to replicate and spread more effectively in susceptible hosts. In fact, CPV-2a, 2b and 2c have reappeared in the host range for cats [8] and have increased their own pathogenicity, causing more severe disease with a shorter incubation period; moreover, the new virus types are shed in the faeces at much higher titres, and a lower virus dose seems to be required for efficient infection [9].

Currently, the original antigenic type 2 is present only in commercial vaccines, and the virus types 2a, 2b and 2c are variously distributed in the canine population worldwide. Numerous scientific papers have reported the frequencies of the different CPV-2 variants in several geographic areas [10]. Epidemiological surveys regarding the distribution of the CPV-2 variants in different countries have shown that CPV-2a is the predominant variant in most of Asia and in European countries, and is the only variant reported in New Zealand. The CPV-2b variant was found to be the predominant antigenic variant in Ireland, the UK, the U.S.A., African countries, several Asian countries and Australia [11]. The CPV-2c variant has mainly been found in European countries and South America, and it has recently been detected in the Australian dog population [12].

In Italy, CPV-2a appeared to be the predominant variant maintaining its prevalence on the others over the time [13–15]. In recent decades, a nearly complete substitution of CPV-2b by CPV-2c has been observed [9, 16] although, despite the initial and sudden peak of detections [17], CPV-2c was the least frequently sequenced variant during the period of the study. A notable difference at the level of local geographic areas has been observed in the distribution of the CPV-2b variant in Italy, with its absence in Sicily [18], and its dominant prevalence in Sardinia [19].

The typing of the CPV-2 variants is commonly based on the different amino acids observed in residue 426 of the VP2 protein (Asn in CPV-2a, Asp in CPV-2b and Glu in CPV-2c), although other specific amino acid changes in VP2 residues have been observed. The CPV-2a and CPV-2b variants showing amino acid change 297 Ser → Ala have been designated as the “new CPV-2a” and “new CPV-2b” [20, 21]; viruses showing a 300 Gly → Asp mutation were designated as “CPV-2c(a)” and “CPV-2(b)” [22]. The Italian CPV-2b variants from Sardinia contained additional amino acid substitutions, and 371 Ala→Gly and 418 Ile → Thr were named the “new CPV-2b” [19]. Along with other specific changes, such as 440 Thr → Ala and 324 Tyr → Ile, CPV-2 did not receive any clear taxonomy or common designation despite their global presence. Other authors have proposed that, in the case of the presence of a new site mutation, the virus could be added into sustainable nomenclature as a new sub-variant [23]. Although not all scientists are in agreement with the virus nomenclature which is somewhat confusing, references to CPV-2a, 2b and 2c are prevalent in the literature. Some of these mutations have changed the antigenic properties of the capsid and have reached high frequencies in viral populations, but the biological consequences of the majority of these changes are, for the most part, unknown [24]. The CPV-2 evolution is ongoing, but the dynamics of the spread and the variation of the virus have recently changed. While the emergence of CPV-2 has been characterised by global viral spread, the endemic phase of the disease has been characterised by geographical genetic differentiation [25] with CPV-2 capsid variants which appear to circulate predominantly locally [26]. The evolution of this virus has raised questions regarding the efficacy of some vaccines; therefore, an understanding of the variation is required.

This study focused on VP2 sequence analysis and the phylodynamics of CPV-2 which were detected in dogs showing clinical signs of gastroenteritis collected in Italy from 1994 to 2017.

## Results

### Study group

The signalment data of the 123 dogs included in this study which tested positive for CPV-2 DNA are summarised in Table 1. The median age of the dogs was 3 months (range 1 month – 10 years). The dogs were of different breeds and of both sexes. Ninety-four out of the 123 (76.4%) dogs came from the mainland of Northern and Central Italy (only two came from Southern Italy, the Basilicata and Apulia regions, respectively) and 29/123 (23.6%) dogs were sampled in the insular area of Italy (the island of Sicily in Southern Italy) in 2009–2010. Forty out of the 123 (32.5%) dogs were vaccinated whereas 57/123 (46.3%) dogs were unvaccinated or incompletely vaccinated; the vaccination status of the remaining 26/123 (21.1%) dogs was unknown.

**Table 1:** Descriptive statistics of the dogs included in the study population

	Dogs positive to CPV-2 DNA *n* = 123 (%)	CPV-2a *n* = 56 (%)	CPV-2b *n* = 18 (%)	CPV-2c *n* = 49 (%)	*P* value
Year of sampling					
1994	1 (0.8)	1 (1.8)	0	0	< 0.0001
1995	5 (4.1)	2 (3.6)	3 (16.7)	0	
1996	2 (1.6)	1 (1.8)	1 (5.6)	0	
1997	1 (0.8)	1 (1.8)	0	0	
1998	2 (1.6)	2 (3.6)	0	0	
1999	4 (3.3)	4 (7.1)	0	0	
2000	7 (5.7)	6 (10.7)	0	1 (2)	
2001	4 (3.3)	3 (5.4)	0	1 (2)	
2002	2 (1.6)	2 (3.6)	0	0	
2003	1 (0.8)	1 (1.8)	0	0	
2005	1 (0.8)	1 (1.8)	0	0	
2006	4 (3.3)	2 (3.6)	0	2 (4.1)	
2007	5 (4.1)	3 (5.4)	0	2 (4.1)	
2008	8 (6.5)	7 (12.5)	0	1 (2)	
2009	37 (30.1)	8 (14.3)	3 (16.7)	26 (53.1)	
2010	7 (5.7)	3 (5.4)	3 (16.7)	1 (2)	
2011	4 (3.3)	4 (7.1)	0	0	
2012	10 (8.1)	3 (5.4)	0	7 (14.3)	
2013	4 (3.3)	0	1 (5.6)	3 (6.1)	
2014	4 (3.3)	2 (3.6)	0	2 (4.1)	
2015	2 (1.6)	0	1 (5.6)	1 (2)	
2016	4 (3.3)	0	3 (16.7)	1 (2)	
2017	4 (3.3)	0	3 (16.7)	1 (2)	
Sex					
Male	61 (49.6)	26 (46.4)	9 (50)	26 (53.1)	0.7866
Female	37 (30.1)	18 (32.2)	4 (22.2)	15 (30.6)	
NA	25 (20.3)	12 (21.4)	5 (27.8)	8 (16.4)	
Age					
< 1	102 (82.9)	46 (82.1)	15 (83.3)	41 (83.7)	0.1496
1–5	4 (3.3)	3 (5.4)	0	1 (2)	
≥ 6	3 (2.4)	2 (3.6)	1 (5.6)	0	
NA	14 (11.4)	5 (8.9)	2 (11.1)	7 (14.3)	
Breed					
Pure breeds	74 (60.2)	37 (66.1)	12 (66.6)	25 (51)	0.3228
Mixed	35 (28.4)	13 (23.2)	5 (27.8)	17 (34.7)	
NA	14 (11.4)	6 (10.7)	1 (5.6)	7 (14.3)	
Origin					
Piedmont	1 (0.8)	0	1 (5.6)	0	0.0001
Veneto	4 (3.3)	4 (7.1)	0	0	
Friuli-Venezia Giulia	1 (0.8)	0	1 (5.6)	0	
Emilia Romagna	80 (65.0)	42 (75)	15 (83.3)	23 (46.9)	
Tuscany	3 (2.4)	2 (3.6)	1 (5.6)	0	
Lazio	1 (0.8)	1 (1.8)	0	0	
Marche	1 (0.8)	0	0	1 (2)	
Abruzzo	1 (0.8)	1 (1.8)	0	0	
Basilicata	1 (0.8)	1 (1.8)	0	0	
Apulia	1 (0.8)	0	0	1 (2)	
Sicily	29 (23.6)	5 (8.9)	0	24 (49)	
Vaccination					
Completely vaccinated	40 (32,5)	21 (37.5)	5 (27.8)	14 (28.6)	0.6799
Incompletely vaccinated	4 (3.3)	2 (3.6)	1 (5.6)	1 (2)	
Unvaccinated	53 (43.1)	23 (41.1)	9 (50)	21 (42.9)	
NA	26 (21.1)	10 (17.8)	3 (16.6)	13 (26.5)	

Data are given as number of dogs and (% of dogs on the total number of dogs included in the group). The data not available (in grey) were not statistically analysed. Significance was set at *P* < 0.05. n: total number of dogs included in the study group. *NA* data not available

### Typing of CPV-2 and DNA sequence analysis

In order to characterise the CPV-2 detected, the coding VP2 gene sequence of 1745 nts was obtained from all viruses. Based on the 426 amino acid residues of the deduced VP2 protein, 56/123 (45.5%) CPV-2 s were classified as the 2a variant owing to the presence of the AAT codon which codes for the amino acid asparagine, 18/123 (14.6%) viruses belonged to CPV-2b owing to the presence of the amino acid aspartate (codon GAT) and 49/123 (39.8%) were characterised as CPV-2c owing to the occurrence of the amino acid glutamate (codon GAA). The original CPV type 2 was not found.

Nucleotide sequences from the 123 viruses sequenced showed high genetic heterogeneity with 67 nucleotide sequence types (ntSTs) (Additional file 1). The majority (46/67, 68.7%) of the ntSTs grouped one nucleotide sequence while 17/67 (25.4%) ntSTs grouped from 2 to 4 nucleotide sequences. The remaining four ntSTs (4/67, 6%), called ntST04 (CPV-2a), ntST38 (CPV-2b), ntST40 (CPV-2c) and ntST43 (CPV-2c), grouped 8, 11, 11 and 7 nucleotide sequences, respectively.

**Fig. 1:**
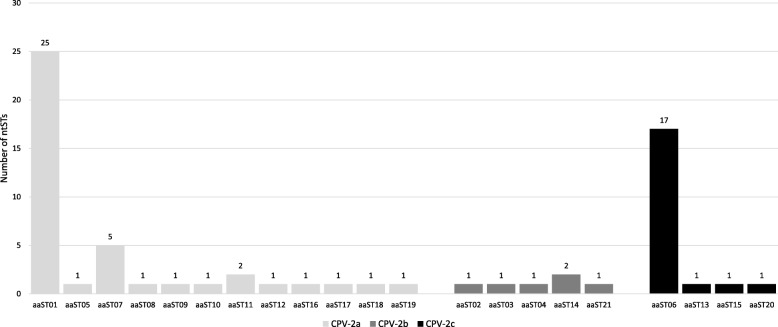
Distribution of amino acid sequence types and nucleotide sequence types in each CPV-2 antigenic variant. aaST: amino acid sequence type. CPV-2: canine parvovirus type 2. ntST: nucleotide sequence type

The VP2 nucleotide sequences were translated into the corresponding amino acid sequences, obtaining 21 amino acid sequence types (aaSTs) (Additional file 1). Five out of the 21 (23.8%) aaSTs included 104/123 (84.5%) nucleotide sequences corresponding to 51/67 (76.1%) ntSTs (Table 2). The remaining 16/21 (76.2%) aaSTs included 19/123 (15.5%) nucleotide sequences corresponding to only one ntST each (16/67 ntSTs, 23.9%). The aaSTs and ntSTs obtained in this study were distributed differently in the three CPV-2 antigenic variants (Additional file 1): CPV-2a grouped 12/21 (57.1%) aaSTs and 41/67 (61.2%) ntSTs; CPV-2b grouped 5/21 (23.8%) aaSTs and 6/67 (8.9%) ntSTs and CPV-2c grouped 4/21 (19.1%) aaSTs and 20/67 (29.9%) ntSTs (Fig. 1).

**Table 2:** Main amino acid sequence types (aaSTs)

	Antigenic variant	Number^a^ and (%) of ntSTs	Number^b^ and (%) of nucleotide sequences
aaST01	CPV-2a	25/67 (37.3%)	39/123 (31.7%)
aaST06	CPV-2c	17/67 (25.4%)	44/123 (35.8%)
aaST07	CPV-2a	5/67 (7.5%)	6/123 (4.9%)
aaST11	CPV-2a	2/67 (3%)	2/123 (1.6%)
aaST14	CPV-2b	2/67 (3%)	13/123 (10.6%)

*aaST* amino acid sequence type, *CPV-2* canine parvovirus type 2, *ntST* nucleotide sequence type

^a^ Expressed as number of nucleotide sequence types (ntSTs) included in the aaST on the total number of ntSTs identified in this study

^b^ Expressed as number of nucleotide sequences included in the aaST on the total number of nucleotide sequences identified in this study

Regarding the 29 viral strains collected in Sicily, 28 were sampled in 2009 and one in 2010. Five out of the 29 (17.2%) viral sequences were CPV-2a and were distinguishable in five ntSTs; the remaining 24/29 (82.8%) sequences were typed as CPV-2c and were distinguishable in six ntSTs (comprising ntST40 and ntST43), prevalently grouped in aaST06 (Additional file 1). No CPV-2b was detected in Sicily. Only one ntST grouping of the Sicilian viral sequences (CPV-2c ntST43) also contained mainland viral sequences (134/2012 KF373602 and 138/2012 MK348086); the remaining ntSTs grouped only Sicilian sequences.

The sequences obtained were aligned and compared to worldwide non-Italian CPV-2 reference strains retrieved from the GenBank database. Nucleotide identity was 99.2–99.9% among the ntSTs obtained, and 98.6–100% among the ntSTs and the worldwide non-Italian CPV-2 reference sequences. Amino acidic similarity was 98.9–99.8% among the aaSTs obtained in this study and 97.9–100% among the aaSTs obtained and the worldwide non-Italian CPV-2 reference sequences.

Several synonymous and non-synonymous substitutions were detected by comparing the sequences. The predicted amino acid substitutions are summarised in Table 3. Seventeen non-synonymous changes were detected in the 21 aaSTs other than the amino acid changes in residue 426 which differentiated the antigenic variants. At least six mutations were very common since they were shared by reference strains detected in various parts of the world: residues 13 (Pro→Ser), 139 (Val → Ile), 297 (Ser → Ala), 324 (Tyr → Ile), 418 (Ile → Thr) and 440 (Thr → Ala). Residue 440 of aaST16, which included the Sicilian CPV-2a X83090/2009 (GenBank ID: KF385388), also showed the change Thr → Ser. Other coding changes have already been described but were less common, such as the 265 Thr → Pro, 371 Ala→Gly, 429 Val → Ile, 517 Asn → Ser and 568 Gly → Ala mutations [13, 19, 27–29]. Instead, the mutations at residues 16 (Arg → Lys), 67 (Arg → Lys), 75 (Glu → Lys), 144 (Glu → Lys) and 356 (Pro→His) were unique and characteristic of the CPV-2a and CPV-2c variants identified in this study. All the aaSTs characterised as the CPV-2a variant in this study showed the amino acid alanine in residue 297 which is considered a sort of marker of the more recent types 2a and 2b (“new CPV-2a” and “new CPV-2b”) [20, 21]. Instead, only the aaSTs 02, 14 and 21 belonging to the CPV-2b variant showed 297Ala while the remaining CPV-2b aaSTs (aaST03 and aaST04) showed the amino acid serine in residue 297. Of the CPV-2b aaSTs, the most recent aaST14 and aaST21 showed unique and distinct mutations with respect to aaST02, aaST03 and aaST04 which were detected in the 1990s: In addition to 297 Ser → Ala, aaST14 and aaST21 showed the mutations 371 Ala→Gly and 418 Ile → Thr, already described in the Italian strains from Sardinia [19].

**Table 3:** Amino acid sequence types (aaSTs) of the Italian CPV strains

aaST of the CPV	13	16	67	75	139	144	265	297	324	356	371	418	426	429	440	517	568	TYPE^a^
aaST01 (*n* = 39)	Pro	Arg	Arg	Glu	Val	Glu	Thr	Ala	Tyr	Pro	Ala	Ile	Asn	Val	Thr	Asn	Gly	CPV-2a
aaST02 (*n* = 1)	–	–	–	–	–	–	–	–	–	–	–	–	Asp	–	–	–	–	CPV-2b
aaST03 (*n* = 2)	–	–	–	–	–	–	Pro	Ser	–	–	–	–	Asp	–	–	–	–	CPV-2b
aaST04 (*n* = 1)	–	–	–	–	–	–	–	Ser	–	–	–	–	Asp	–	–	–	–	CPV-2b
aaST05 (*n* = 1)	–	–	–	–	–	–	–	–	–	–	–	–	–	–	–	Ser	–	CPV-2a
aaST06 (*n* = 44)	–	–	–	–	–	–	–	–	–	–	–	–	Glu	–	–	–	–	CPV-2c
aaST07 (*n* = 6)	–	–	–	–	–	–	–	–	–	–	–	–	–	–	Ala	–	–	CPV-2a
aaST08 (*n* = 1)	–	–	–	–	–	–	–	–	–	–	–	–	–	–	–	–	Ala	CPV-2a
aaST09 (*n* = 1)	–	–	Lys	Lys	–	–	–	–	–	–	–	–	–	–	–	–	–	CPV-2a
aaST10 (*n* = 1)	–	Lys	–	–	–	Lys	–	–	–	–	–	–	–	–	–	–	–	CPV-2a
aaST11 (*n* = 2)	–	Lys	–	Lys	–	–	–	–	–	–	–	–	–	–	–	–	–	CPV-2a
aaST12 (*n* = 1)	Ser	–	–	–	–	–	–	–	–	–	–	–	–	–	–	–	–	CPV-2a
aaST13 (*n* = 1)	–	–	–	Lys	–	–	–	–	–	–	–	–	Glu	–	–	–	–	CPV-2c
aaST14 (*n* = 13)	Ser	–	–	–	–	–	–	–	–	–	Gly	Thr	Asp	–	–	–	–	CPV-2b
aaST15 (*n* = 2)	–	–	–	–	–	–	–	–	–	–	–	–	Glu	Ile	–	–	–	CPV-2c
aaST16 (*n* = 1)	–	–	–	–	–	–	–	–	–	–	–	–	–	–	Ser	–	–	CPV-2a
aaST17 (*n* = 1)	–	–	–	–	–	–	–	–	–	His	–	–	–	–	–	–	–	CPV-2a
aaST18 (*n* = 1)	–	–	–	–	Ile	–	–	–	–	–	–	–	–	–	–	–	–	CPV-2a
aaST19 (*n* = 1)	–	–	–	–	–	–	–	–	Ile	–	–	–	–	–	–	–	–	CPV-2a
aaST20 (*n* = 2)	–	–	–	–	Ile	–	–	–	–	–	–	–	Glu	–	–	–	–	CPV-2c
aaST21 (*n* = 1)	–	–	–	–	–	–	–	–	–	–	Gly	Thr	Asp	–	–	–	–	CPV-2b

Amino acid positions are reported in the first line. In brackets: the number of nucleotide sequences belonging to the aaST. *aaST* amino acid sequence type, *CPV-2* canine parvovirus type 2

^a^ CPV-2 antigenic variant deduced from amino acid residue 426

The results of the DnaSP analyses and the Simpson diversity index (D) value calculated for each CPV-2 antigenic variant are reported in Table 4. As compared to the other variants, CPV-2a showed the highest sequence variability, assessed by the nucleotide diversity Pi (π) and the average number of nucleotide differences (k). Conversely, the CPV-2c variant showed the lowest sequence variability; the same result was obtained for the CPV-2c variant when analysing only the sequences coming from Sicily as well analysing the type 2c sequence data set, excluding the Sicilian ones (Additional file 2). The CPV-2a and 2b types had the lowest and the highest values of D, respectively, but CPV-2b showed the highest a/η rate (the total number of non-synonymous differences on the total number of mutations), demonstrating a higher impact of nucleotide variability on the viral phenotype.

**Table 4:** Summaries of the sequence variability and Simpson’s index of the three CPV-2 antigenic variants

	No. of sequences	Total no. of sites	*S*	η	π	s	a	a/η	k	D
CPV-2a	56	1745	53	55	0.00251 SD 0.00019	42	13	0.23636	4.384	0.02403
CPV-2b	18	1745	16	16	0.00227 SD 0.00061	11	5	0.3125	3.961	0.37255
CPV-2c	49	1745	26	26	0.00132 SD 0.00013	23	3	0.11538	2.301	0.08078
Tot.	123	1745	81	83	0.00329 SD 0.00012	63	20	0.24096	5.747	0.02519

a: total number of non-synonymous differences. a/η: total number of non-synonymous differences on the total number of mutations. CPV-2: canine parvovirus type 2. D: Simpson’s index. k: average number of nucleotide differences. η: total number of mutations. π: nucleotide diversity (average number of nucleotide differences per site) and standard deviation. s: total number of synonymous differences. *S*: number of polymorphic (segregating) sites

**Fig. 2:**
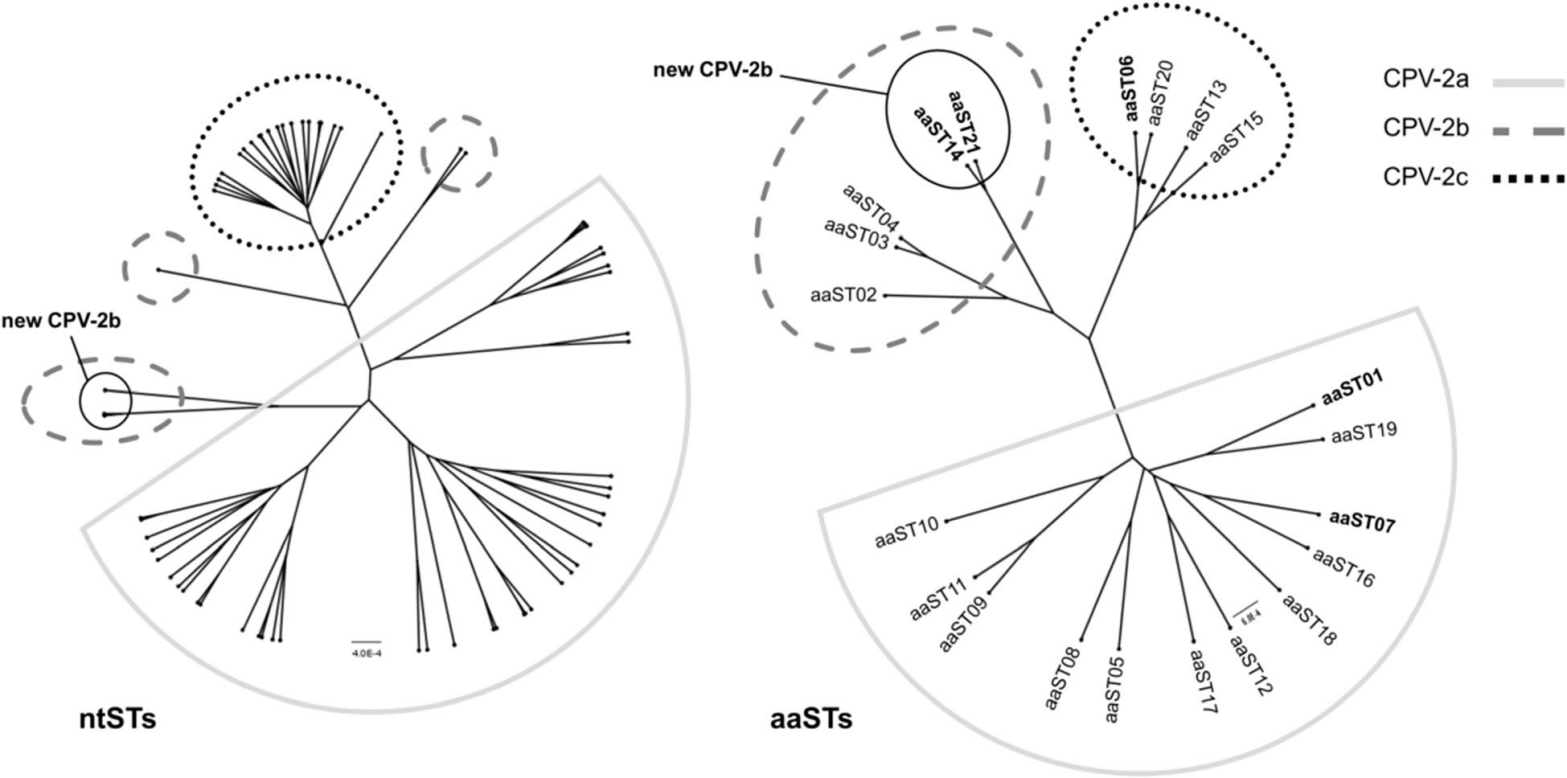
Phylogenetic trees constructed on the VP2 nucleotide and amino acid sequence types obtained in this study. A coalescent constant population tree was estimated for both the ntSTs and aaSTs obtained in this study using BEAST 1.10.4 and BEAGLE 3.1.0 software. The Tamura 93 model with gamma distribution and the Jones-Taylor-Thornton (JTT) model with gamma distribution were used to build the ntST and the aaST phylogenetic trees, respectively. Phylogenetic analysis was carried out using the “strict clock” as a clock model for both nucleotide and amino acid sequence types. The Bayesian Markov chain Monte Carlo (MCMC) chain lengths were 40,000,000 generations, with sampling every 2000 generations. The tree iteration was discharged with 10% of the chains as a burn-in pattern by using a tree annotator, and the resulting MCMC tree was drawn using FigTree software. 1.4.2. Nucleotide STs and aaSTs were represented by points. In the ntST tree, the taxons were represented by points. In the aaST tree, the most important sequence types were in bold

The CPV-2a, CPV-2b and CPV-2c variants formed three well-distinguishable clusters in the amino acid phylogenetic tree constructed with the VP2 sequences obtained in this study (Fig. 2). However, in the phylogenetic tree constructed with the VP2 nucleotide sequences, the CPV-2c viruses formed a separate cluster whereas the CPV-2a and CPV-2b sequences appeared partially intermingled. The sequences corresponding to the most recent new CPV-2b variant (aaST14 and aaST21) formed a clearly distinguishable lineage in both phylogenetic trees and seemed to have an origin close to the CPV-2a viruses in the tree constructed with the nucleotide sequences (Fig. 2). A similar clustering was shown by the phylogenetic tree constructed on the VP2 amino acid sequence types obtained in this study and the worldwide reference sequences (Additional file 3). The aaSTs corresponding to the new CPV-2b appeared to be correlated to a Vietnamese CPV-2b variant identified in 2002 (AB120721) which, however, did not show the amino acid mutations 371 Ala→Gly and 418 Ile → Thr [30].

### Statistical analysis

**Fig. 3:**
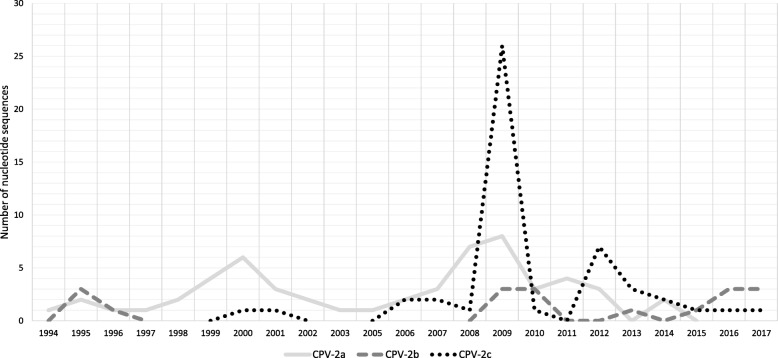
Number of CPV-2 nucleotide sequences belonging to the CPV-2 antigenic variants identified in this study. CPV-2: canine parvovirus type 2

No statistical association was found among the CPV-2 antigenic variants, and sex, age, breed and vaccination status (Table 1). Only the year of sampling and geographical origin were statistically different between the CPV-2a, 2b and 2c variants (*P* <  0.0001 and *P* = 0.0001, respectively). In the 24-year observation period, the frequency of the CPV-2 variants showed rapid oscillation (Fig. 3). The CPV-2a variant was commonly detected from 1994 to 2014, with the exception of 2013, and it was not found from 2015 to 2017. The CPV-2b variant was detected in 1995–1996, together with variant 2a; it was no longer detected from 1997 to 2008, and it reappeared in 2009. From 2009 until 2017, CPV-2b was discovered, although not continuously. The CPV-2c variant was detected for the first time in 2000 and, subsequently, it was not continuously found until 2017 with a peak in 2009 represented by the strains from Sicily.

**Fig. 4:**
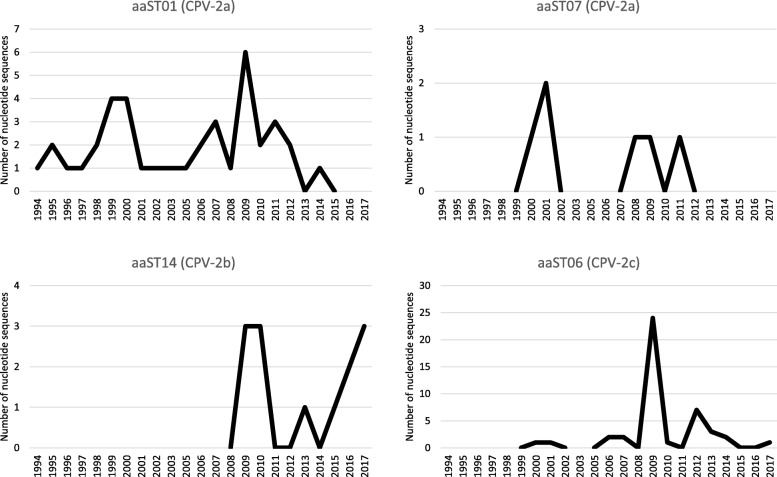
Number of CPV-2 nucleotide sequences belonging to aaST01, aaST06, aaST07 and aaST14. aaST: amino acid sequence type. CPV-2: canine parvovirus type 2

Regarding the distribution of the aaSTs over time, the majority of them appeared for just 1 year, at most 2 years, and were then no longer detected. Four aaSTs were an exception (Fig. 4): 1) aaST01, the predominant CPV-2a amino acid sequence type with 39/56 (66.1%) nucleotide sequences, was detected from 1994 to 2014; 2) aaST07 (CPV-2a) was detected in 2000–2001, 2008–2009 and 2011; 3) aaST14, the predominant CPV-2b amino acid sequence type with 13/18 (72.2%) nucleotide sequences, was detected for the first time in 2009 and it was also detected in 2010, 2013 and 2015–2017 and 4) aaST06, the predominant CPV-2c amino acid sequence type with 44/49 (89.8%) nucleotide sequences was detected for the first time in 2000 and it was not continuously detected until 2017 with a peak in 2009, represented by the Sicilian viruses.

## Discussion

The present study provides an update regarding the distribution of the three CPV-2 variants in Italy from 1994 to 2017 and the evolution dynamics of the CPV-2 population in order to add new data regarding the diffusion and evolution of the three viral types 2a, 2b and 2c over a long time frame. Although not all scientists are in agreement with the virus nomenclature, the use of the terms CPV-2a, −2b and -2c is prevalent in the literature and, therefore, this terminology was used throughout the text.

During the 24-year study period, 123 diarrhoeic dogs testing positive for CPV-2 DNA were included in the study. For the most part, the dogs included were young (median age 3 months), reflecting the tropism of CPV-2 in dogs [3]. The majority of the dogs (76.4%) came from the mainland of Italy and 23.6% of the dogs came from the island of Sicily in Southern Italy.

Approximately a third (32.5%) of the dogs involved had been vaccinated, raising doubts regarding the correct application of the vaccination protocol or the ability of the vaccine to confer protective immunity. Previous studies had reported the occurrence of parvovirus infection in vaccinated dogs; the main cause of vaccination failure is the interfering role of maternal antibodies, although the evolution of the virus also raises questions regarding the efficacy of some vaccines and the necessity of updating the vaccine according to the new variants currently circulating in the canine population [4, 10, 31, 32]. Other studies have demonstrated a high prevalence of non-responders, above all in certain breeds [33]. The vaccination protocol of the dogs tested in this study was unknown; however, since they were, for the most part, puppies, the potential cause of vaccination failure could have been interaction with maternally derived antibodies preventing the onset of effective immunity. Furthermore, pre-exposure to CPV-2 prior to vaccination could have been a contributing factor in some cases. No statistical relationship was found between the vaccination status, and the antigenic variants or aaSTs, excluding the fact that a specific antigenic variant or a specific amino acid sequence type may have eluded the vaccine-type immune response. A potential implication of the elevated evolution rate for vaccination efficacy was hypothesised. Canine parvovirus type 2 has the tendency of accumulating mutations, and the mismatch between vaccines and wild strains may have contributed to increasing the risk of an outbreak in the canine population; however, to date, a possible immune escape of CPV-2 variants has not yet been conclusively proven, and it has not been possible to reach any final conclusions [34, 35].

Canine parvovirus type 2a was the predominant variant detected in the dogs in this study, representing 45.5% of the viral sequences analysed, confirming previous data reported in Italy from 2008 to 2015 by Tucciarone and collaborators [15]. Canine parvovirus type 2a was continuously identified during the first 20 years of the study period, confirming its predominance in the Italian canine population until the first decade of the 2000s [13, 14]. This variant showed the highest diversity and sequence variability which was reflected by the largest number of ntSTs (41/67, 61.2%) and aaSTs (12/21, 57.1%) belonging to it. Furthermore, the Simpson diversity index, which is a measurement of diversity, was lower for type 2a than for the other CPV-2 variants. This result can allow hypothesising that the high diffusion in the canine population characterising the CPV-2a variant influenced the sequence variability of this variant, leading the virus to accumulate numerous nucleotide and amino acid mutations over time. In particular, of the 12 CPV-2a amino acid sequence types, aaST01 and aaST07 showed the highest fitness advantage. Amino acid ST01 was the most recurrent aaST, grouping 39 nucleotide sequences (25 ntSTs) identified from 1994 to 2014. Amino acid ST07, characterised by the amino acid mutation 440 Thr → Ala, grouped six nucleotide sequences (five ntSTs) detected in this study from 2000 to 2011. The 440 residue is located in the GH loop, a region exposed on the surface of the capsid and forms the 22 Å threefold spike; the greatest variability between parvoviruses was observed in this antigenic region [36]. This mutation was found for the first time in Italy in 2000 in the 2a variant [27]; it was then reported worldwide. The recent CPV-2 variants detected in various parts of the world have the 440 Thr → Ala substitution; in several geographic areas, this change was restricted to the CPV-2c variant [37, 38] while, in other countries, it was also detected in both the CPV-2a and 2b variants [39]. In this study, the 440 Thr → Ala change was limited to the CPV-2a variant. In aaST16, which includes just one CPV-2a sequence (X83090/2009, GenBank ID: KF385388), the 440 Thr → Ser change, which was previously described in one Italian FPV strain [40] and in Ecuadorian CPV-2 strains where it was shown in all variants 2a, 2b and 2c [41] was detected. Another amino acid change involving residue 440 (Thr → Pro) has recently been reported in Sardinia, Italy [19], confirming the high variability of the region as well as the fact that residue 440 was under positive selective pressure.

The CPV-2b variant, detected in 18/123 dogs sampled from the mainland of Italy, was the least prevalent variant identified in this study. It was detected in the first years of this study but it was no longer detected between 1997 and 2008. Starting from 2009, CPV-2b was identified until 2017 in the mainland of Italy. This reappearance such a long time after its last detection, associated with its permanence in the canine population in subsequent years, could suggest that a new CPV-2b emerged. In fact, in 2009, a CPV-2b variant with a unique VP2 amino acid sequence corresponding to aaST14 was identified. Canine parvovirus type 2b aaST14 was characterised by the amino acid mutations 13 Pro→Ser, 371 Ala→Gly and 418 Ile → Thr in the VP2 deduced protein. Amino acid ST14 grouped 13 nucleotide sequences (2 ntSTs), and it was the only CPV-2b aaST identified in this study from 2009 to 2017 with the exception of one CPV-2b variant detected in 2016 which alone constituted aaST21. The VP2 protein sequence of aaST21 was identical to aaST14 except for the lack of a mutation in position 13. Canine parvovirus type 2b strains with analogous amino acid changes were detected in several parts of Italy during the same period (2008–2015) [15, 19], but they were not reported in other parts of the world. The amino acid mutations in residues 371 and 418 of the VP2 protein probably gave an adaptive advantage to the “new CPV-2b” and allowed it to spread and stabilise in the canine population. The 418 Ile → Thr substitution located in the GH loop of VP2 was reported for CPV-2a in Italy [21] and, in addition to Italy, it was also reported in Asian countries where it was a common mutation shown by Korean and Japanese strains [42, 43]. Additional studies are needed to understand how the amino acid mutations 371 Ala→Gly and 418 Ile → Thr could affect the replicative efficiency of this virus, and whether this “new CPV-2b” originated from adaptive mutations of viruses already present in the Italian territory or whether it could have been introduced into the Italian territory by means of dogs being transported. Since, to date, this “new CPV-2b” has been detected only in Italy, it is probably the result of local differentiation. From the sequence analysis, CPV-2b was more genetically stable than CPV-2a but showed the highest fraction of non-synonymous mutations, highlighting a significant phenotypic effect of the accumulated mutations over time. A similar situation was observed in Australia where CPV-2b subtypes have continued to evolve at a relatively rapid rate in recent years [11].

The number of viruses belonging to the CPV-2c variant identified in this study (49/123, 39.8%) was comparable to that of CPV-2a. This variant was detected for the first time in 2000 and was not continuously detected until the end of the study period. Nevertheless, CPV-2c appeared to be the least variable variant with only four aaSTs grouping 20 ntSTs of which 17 were included in aaST06. The low variability of this variant was confirmed by the high number of nucleotide sequences (44/49) referring to aaST06, identified from 2000 to 2017, both on the mainland and in insular areas, which therefore proved to have a high fitness in replicating and spreading in the canine population under study. Half of the CPV-2c sequences analysed (24/49) were sampled in Sicily in 2009, and 22 belonged to aaST06. From these data, it could be interpreted that the elevated number of homogeneous viral sequences coming from Sicily in 2009 may have altered the calculated variability of the CPV-2c variant. Instead, excluding all the viral sequences identified in dogs from Sicily, the CPV-2c variant was still the least variable. This was probably because aaST06 was highly widespread and also predominant on the mainland.

In Sicily, CPV-2c was the predominant variant (24/29), followed by CPV-2a with five identified viruses; CPV-2b was apparently not spread in dogs of the insular territory. The Sicilian viral strains analysed in the present study were collected in 2009–2010 while CPV-2b reappeared on the mainland of Italy in 2009; on the basis of this trend, the subsequent introduction and spread of CPV-2b in Sicily was conceivable. The latter hypothesis has to be excluded because a recent survey carried out in Sicily from 2009 to 2015 reported a high prevalence of the 2c variant in the absence of CPV-2b [18]. This significant difference between Sicily and the Italian mainland probably depended on the different geographical basis, illustrating different variant/subtypes among the areas analysed within the same country. Similarly, in Sardinia, the other major Italian island, a recent survey has shown an additional diverse epidemiological situation with a prevalence of CPV-2b [19].

From the data analysed, a representative aaST grouping the majority of the ntSTs was found for each CPV-2 antigenic variant. For CPV-2a, the predominant aaST was number 01; for CPV-2b, it was aaST14 and, for CPV-2c, it was aaST06, both on the mainland and in the insular areas of Italy. Taken together, these three aaSTs grouped 44/67 ntSTs and 96/123 VP2 nucleotide sequences identified in this study. The viruses showing the predominant amino acid sequence would probably be more adapted to replicate in the host and fit the environmental conditions, allowing them to spread and last over time in the canine population. All other aaSTs, being less adapted to spreading in the canine population, disappeared quickly after their emergence. In fact, with the exception of CPV-2a aaST07 grouped five ntSTs and six nucleotide sequences detected from 2000 to 2011; the remaining 17/21 aaSTs grouped 21/123 nucleotide sequences belonging to 18/67 ntSTs and were identified for no more than 1 or 2 years in this study.

Sequence analysis revealed several coding changes, some of them involving residues located on the capsid surface, where important antigenic sites are located. Single mutations have been shown to affect the binding of monoclonal antibodies with consequent CPV-2 evasion of humoral immunity [44]. The high variability of the surface antigenic sites may also be responsible for variation in host specificity as indicated by the overlap of the transferrin receptor (TfR) binding site with the antigenic sites [45]. Although the functional impact of the majority of mutations has yet to be ascertained, the present study indicated active evolution of these strains.

## Conclusions

From the data herein analysed, CPV-2a was the prevalent variant circulating in the canine population of mainland Italy from 1994 to the first decade of the 2000s. On the contrary, CPV-2b and 2c were not detected or were detected only sporadically until the 2000s, and have been successively identified with increased frequency. The CPV-2a variant was characterised by the highest genetic variability; instead, CPV-2c was characterised by notable stability with a predominant amino acid profile. Finally, from its remergence in recent years, CPV-2b has shown a new and distinctive profile of the VP2 protein, characterised by Asn 517-to-Ser and Ala 371-to-Gly changes. Additional studies are warranted to confirm an increased diffusion of the variants CPV-2b and 2c at the expense of the CPV-2a on the Italian mainland and in many other countries [11, 37]; monitoring of the evolution and spread of the new CPV-2 variants should be a key aim of ongoing research.

## Methods

### Samples

One-hundred and twenty-three diarrhoeic dogs which tested positive to CPV-2 DNA using a qualitative polymerase chain reaction (PCR) assay [46] carried out on faecal samples collected from 1994 to 2017 were retrospectively included in this study. Faecal samples were tested at the Service of Clinical Pathology (Department of Veterinary Medical Sciences – DIMEVET, University of Bologna, Italy) and at the Istituto Zooprofilattico Sperimentale della Sicilia “A. Mirri” (Palermo, Italy). Signalment data (year of sampling, sex, age, breed and geographical origin) and vaccination status of the dogs which tested positive were retrieved from medical records.

### Molecular characterisation of the CPV-2 strains

Viral DNA was extracted from faecal samples using the NucleoSpin Tissue Mini Kit (Macherey-Nagel, Düren, Germany) according to the manufacturer’s instructions. The VP2 gene of CPV-2 was amplified as previously described by Battilani and collaborators [47], and amplicons of expected size (1745 nts) were directly sequenced using the Sanger method, using both forward and reverse primers, and a third internal primer, primer 41 (5′- GCC CTT GTG TAG ACG C -3′) [48].

The nucleotide sequences obtained were aligned using the CLUSTAL W method implemented in BioEdit sequence alignment editor version 7.2.5 [49]. and were translated into amino acid sequences. The antigenic variants (CPV-2a, 2b and 2c) were deduced from the sequences based on amino acid residue 426. The sequences were grouped on the basis of nucleotide heterogeneity into different ntSTs which shared unique genotypes and on the basis of amino acid heterogeneity in different aaSTs which shared unique deduced protein sequences. The ntSTs and aaSTs obtained were compared to 175 worldwide non-Italian CPV-2 reference sequences (Additional file 4) available from the GenBank database (https://www.ncbi.nlm.nih.gov/genbank/), and the degree of similarity among the sequences at both the nucleotide and the amino acid levels was determined using BioEdit software.

In order to estimate the genetic diversity and the sequence variability of the sequence data, a variety of statistical analyses regarding nucleotide polymorphism and sequence variability were assessed using DnaSP version 6 [50]. Statistical analysis was carried out on the antigenic variant (CPV-2a, 2b and 2c) subpopulations. The following parameters were estimated: the number of polymorphic sites (*S*), π (which was the average number of nucleotide differences per site between two sequences) and its standard deviation, the total number of mutations (η), the total number of synonymous (s) and non-synonymous (a) differences, the a/η rate and k. In addition, D was calculated as a measure of population diversity:

$$ \mathrm{D}=\frac{\sum \mathrm{n}\;\left(\mathrm{n}\hbox{-} 1\right)}{\mathrm{N}\;\left(\mathrm{N}\hbox{-} 1\right)} $$

where N is the total number of all sequences and n is the number of sequences for each nucleotide sequence type (ntST). The value of D ranged from 0 to 1. A D value of 0 represented infinite diversity and 1 corresponded to no diversity, that is, the lower the value of D, the greater the species diversity [51].

### Phylogenetic analysis

The viral sequences obtained in this study were analysed using BEAST version 1.10.4 and BEAGLE version 3.1.0 software [52–54] using a strict clock model with a coalescent constant population. Tamura-Nei and Jones-Taylor-Thornton (JTT) models with gamma distribution were used to build the ntST and the aaST phylogenetic trees, respectively. In addition, a set of protein sequences including 175 worldwide non-Italian CPV-2 amino acid reference sequences (Additional file 4) was analysed using a strict clock model with a coalescent constant population and a Jones-Taylor-Thorton (JTT) model with gamma distribution. The Bayesian Markov chain Monte Carlo (MCMC) chain lengths were 40,000,000 generations, with sampling every 2000 generations. The tree iteration was discharged with 10% of the chains as a burn-in pattern by using a tree annotator, and the resulting MCMC tree was drawn with FigTree software (v1.4.2) (http://tree.bio.ed.ac.uk/software/figtree/).

### Statistical analysis

Signalment data and vaccination status were grouped as reported in Table 1 and were evaluated using standard descriptive statistics. In particular, the distribution of the three CPV-2 antigenic variants and the aaSTs detected during the 24-year sampling period were compared. The viruses detected were grouped on the basis of the antigenic variant (CPV-2a, 2b and 2c), and clinical data were analysed using the Chi-squared (χ2) test. The results were considered significant when P was < 0.05. Statistical analyses were carried out using statistical software (MedCalc Statistical Software version 18.5, MedCalc Software bvba, Ostend, Belgium).

## Supplementary information

**Additional file 1.** Canine parvovirus type 2 (CPV-2) antigenic variants, amino acid sequence types (aaSTs), nucleotide sequence types (ntSTs) and nucleotide sequences obtained in this study. Sequences are reported with: year of identification, lab ID number, antigenic variant and GenBank accession number.

**Additional file 2 **Sequence variability and Simpson’s index of the canine parvovirus type 2c (CPV-2c) sequences without Sicilian strains and of the CPV-2c Sicilian sequences. a: total number of non-synonymous differences. a/η: total number of non-synonymous differences on the total number of mutations. CPV-2: canine parvovirus type 2. D: Simpson’s index. k: average number of nucleotide differences. η: total number of mutations. π: nucleotide diversity (average number of nucleotide differences per site) and standard deviation. s: total number of synonymous differences. *S*: number of polymorphic (segregating) sites.

**Additional file 3.** The unrooted phylogenetic tree constructed on VP2 amino acid sequence types obtained in this study and worldwide non-Italian canine parvovirus type 2 (CPV-2) amino acid reference sequences. A coalescent constant population tree was estimated using BEAST 1.10.4 and BEAGLE 3.1.0 software utilising a strict clock model with a coalescent constant population. The Jones-Taylor-Thornton (JTT) model with gamma distribution was used to build the phylogenetic trees. Phylogenetic analysis was carried out using the “strict clock” as a clock model; 175 worldwide non-Italian CPV-2 amino acid reference sequences (Additional file 4) were included in the analysis. The Bayesian Markov chain Monte Carlo (MCMC) chain lengths were 40,000,000 generations, with sampling every 2000 generations. The tree iteration was discharged with 10% of the chains as a burn-in pattern by using a tree annotator and the resulting MCMC tree was drawn using FigTree software 1.4.2. In blue, the CPV-2a aaSTs obtained in this study. In red, the CPV-2b aaSTs obtained in this study. In green the CPV-2c aaSTs obtained in this study.

**Additional file 4.** List of the 175 worldwide non-Italian canine parvovirus type 2 (CPV-2) reference sequences retrieved from the GenBank database and used for sequence analysis. Sequences are reported with: year of identification, acronym of the nation, antigenic variant and GenBank accession number.

## Data Availability

The datasets supporting the conclusions of this article are included within the article (and its additional files). The nucleotide sequences generated during the current study are available in the GenBank repository under accession numbers: KF373568-KF373611, KF385382-KF385391 and MK348065-MK348125. The sequences AF306445-AF306450, AF393506 and AF401519 originated in previous studies [13, 27].

## Abbreviations

a: Total number of non-synonymous differences
a/η: Total number of non-synonymous differences on the total number of mutations
aaST: Amino acid sequence type
Ala: Alanine
Arg: Arginine
Asn: Asparagine
Asp: Aspartic acid
CPV-2: Canine parvovirus type 2
D: Simpson’s index
FPV: Feline panleukopenia virus
Glu: Glutamic acid
Gly: Glycine
His: Histidine
Ile: Isoleucine
JTT: Jones-Taylor-Thornton
k: Average number of nucleotide differences
Lys: Lysine
MCMC: Markov chain Monte Carlo
MEV: Mink enteritis virus
nts: Nucleotides
ntST: Nucleotide sequence type
PCR: Polymerase chain reaction
Pro: Proline
RaPV: Raccoon parvovirus
*S*: Number of polymorphic sites
s: Total number of synonymous differences
Ser: Serine
Thr: Threonine
Val: Valine
VP2: Viral protein 2
η: Total number of mutations
π: Nucleotide diversity
χ2: Chi-squared
