# Supplementary material for: Molecular epidemiology of canine parvovirus type 2 in Italy from 1994 to 2017: recurrence of the CPV-2b variant
Source: BMC Vet Res. 2019 Nov 4;15:393. doi: 10.1186/s12917-019-2096-1 (PMC6829998; doi:10.1186/s12917-019-2096-1)
Supplement: Supplementary file 1 — Additional file 1. Canine parvovirus type 2 (CPV-2) antigenic variants, amino acid sequence types (aaSTs), nucleotide sequence types (ntSTs) and nucleotide sequences obtained in this study. Sequences are reported with: year of identification, lab ID number, antigenic variant and GenBank accession number. [file 12917_2019_2096_MOESM1_ESM.docx]

CPV-2 nucleotide sequence types (ntSTs) and nucleotide sequences obtained in this study.

Sequences are named with: year of identification, lab ID number, antigenic variant and GenBank (https://www.ncbi.nlm.nih.gov/genbank/) accession number.

| **ntST number** | **CPV-2 nucleotide sequences** |
| --- | --- |
| **ntST01** | 1994_0584_CPV-2a_ AF306446 1995_0589_CPV-2a_ MK348065 |
| **ntST02** | 1995_0598_CPV-2b_ KF373568 |
| **ntST03** | 1995_0603_CPV-2b_ KF373569 1995_0616_CPV-2b_AF306449 |
| **ntST04** | 1995_0618_CPV-2a_AF306447 1997_0660_CPV-2a_MK348066 1998_0679_CPV-2a_MK348067 1999_0687_CPV-2a_MK348068 2000_0697_CPV-2a_MK348069 2009_0212_CPV-2a_MK348076 2009_0272_CPV-2a_MK348077 2014_0577_CPV-2a_MK348093 |
| **ntST05** | 1996_0632_CPV-2a_AF306445 |
| **ntST06** | 1996_0637_CPV-2b_AF306450 |
| **ntST07** | 1998_0677_CPV-2a_AF306448 |
| **ntST08** | 1999_0684_CPV-2a_KF373570 |
| **ntST09** | 1999_0685_CPV-2a_KF373571 |
| **ntST10** | 1999_0689_CPV-2a_KF373572 |
| **ntST11** | 2000_0690_CPV-2a_KF373573 |
| **ntST12** | 2000_0695_CPV-2c_AF401519 2006_0586_CPV-2c_MK348073 2017_0847_CPV-2c_MK348103 |
| **ntST13** | 2000_0699_CPV-2a_AF393506 |
| **ntST14** | 2000_0701_CPV-2a_KF373574 2000_0704_CPV-2a_MK348070 2000_0706_CPV-2a_MK348071 |
| **ntST15** | 2001_0707_CPV-2c_KF373575 |
| **ntST16** | 2001_0709_CPV-2a_KF373576 2001_0710_CPV-2a_MK348072 |
| **ntST17** | 2001_0714_CPV-2a_KF373577 |
| **ntST18** | 2002_0305_CPV-2a_KF373578 |
| **ntST19** | 2002_0312_CPV-2a_KF373579 |
| **ntST20** | 2003_0581_CPV-2a_KF373580 2009_0314_CPV-2a_MK348078 |
| **ntST21** | 2005_1805_CPV-2a_KF373581 |
| **ntST22** | 2006_0258_CPV-2a_KF373582 |
| **ntST23** | 2006_0691_CPV-2a_KF373583 |
| **ntST24** | 2006_1401_CPV-2c_KF373584 |
| **ntST25** | 2007_0236_CPV-2a_KF373585 2008_0059_CPV-2a_MK348075 |
| **ntST26** | 2007_0241_CPV-2c_KF373586 |
| **ntST27** | 2007_0733_CPV-2a_KF373587 2007_0996_CPV-2a_MK348074 |
| **ntST28** | 2007_1447_CPV-2c_KF373588 |
| **ntST29** | 2008_0060_CPV-2a_KF373589 |
| **ntST30** | 2008_0096_CPV-2a_KF373590 |
| **ntST31** | 2008_0097_CPV-2a_KF373591 |
| **ntST32** | 2008_0329_CPV-2a_KF373592 |
| **ntST33** | 2008_0331_CPV-2c_KF373593 |
| **ntST34** | 2008_0332_CPV-2a_KF373594 |
| **ntST35** | 2008_0947_CPV-2a_KF373595 |
| **ntST36** | 2009_0109_CPV-2a_KF373596 |
| **ntST37** | 2009_0326_CPV-2c_MK348079 2009_0800_CPV-2c_KF373597 |
| **ntST38** | 2009_0461_CPV-2b_KF373599 2009_0462_CPV-2b_MK348080 2009_0463_CPV-2b_MK348081 2010_0115_CPV-2b_MK348082 2010_0120_CPV-2b_MK348083 2010_0121_CPV-2b_MK348084 2013_0392_CPV-2b_MK348090 2015_1151_CPV-2b_MK348097 2016_1251_CPV-2b_MK348100 2016_1305_CPV-2b_MK348101 2017_0934_CPV-2b_MK348106 |
| **ntST39** | 2009_X8309_CPV-2a_KF385390 |
| **ntST40** | 2009_X9896_CPV-2c_MK348107 2009_X23782_CPV-2c_MK348108 2009_X60282_CPV-2c_MK348113 2009_X60289_CPV-2c_MK348114 2009_X60298_CPV-2c_MK348115 2009_X60301_CPV-2c_MK348116 2009_X60304_CPV-2c_MK348117 2009_X60318_CPV-2c_MK348118 2009_X60384_CPV-2c_MK348121 2009_X78380_CPV-2c_KF385383 2009_X78391_CPV-2c_MK348125 |
| **ntST41** | 2009_X25835_CPV-2c_KF385385 2009_X25965_CPV-2c_MK348109 2009_X38718_CPV-2c_MK348110 |
| **ntST42** | 2009_X29451_CPV-2a_KF385391 |
| **ntST43** | 2009_X45361_CPV-2c_MK348111 2009_X60327_CPV-2c_MK348119 2009_X60339_CPV-2c_MK348120 2009_X60414_CPV-2c_MK348122 2009_X78184_CPV-2c_MK348123 2012_0134_CPV-2c_KF373602 2012_0138_CPV-2c_MK348086 |
| **ntST44** | 2009_X60203_CPV-2c_KF385382 2009_X60230_CPV-2c_MK348112 |
| **ntST45** | 2009_X60406_CPV-2c_KF385384 |
| **ntST46** | 2009_X60416_CPV-2c_KF385386 2009_X78375_CPV-2c_MK348124 |
| **ntST47** | 2009_X60666_CPV-2a_KF385387 |
| **ntST48** | 2009_X83090_CPV-2a_KF385388 |
| **ntST49** | 2010_0118_CPV-2c_KF373598 2012_0263_CPV-2c_MK348087 2013_0570_CPV-2c_MK348092 2014_0608_CPV-2c_MK348094 |
| **ntST50** | 2010_0409_CPV-2a_KF373611 |
| **ntST51** | 2010_0540_CPV-2a_KF373610 |
| **ntST52** | 2010_X987_CPV-2a_KF385389 |
| **ntST53** | 2011_0042_CPV-2a_KF373600 2011_0055_CPV-2a_MK348085 |
| **ntST54** | 2011_0053_CPV-2a_KF373605 |
| **ntST55** | 2011_0088_CPV-2a_KF373601 |
| **ntST56** | 2012_0149_CPV-2a_KF373606 |
| **ntST57** | 2012_0151_CPV-2a_KF373603 |
| **ntST58** | 2012_0294_CPV-2c_KF373604 |
| **ntST59** | 2012_0304_CPV-2a_KF373607 |
| **ntST60** | 2012_0334_CPV-2c_KF373608 2012_0340_CPV-2c_MK348088 2012_0341_CPV-2c_MK348089 |
| **ntST61** | 2013_0397_CPV-2c_KF373609 |
| **ntST62** | 2013_0538_CPV-2c_MK348091 |
| **ntST63** | 2014_0673_CPV-2c_MK348095 |
| **ntST64** | 2014_0791_CPV-2a_MK348096 |
| **ntST65** | 2015_1154_CPV-2c_MK348098 2016_1203_CPV-2c_MK348099 |
| **ntST66** | 2016_1418_CPV-2b_MK348102 |
| **ntST67** | 2017_0850_CPV-2b_MK348104 2017_0851_CPV-2b_MK348105 |

CPV-2 amino acid sequence types (aaSTs) and nucleotide sequence types (ntSTs) obtained in this study.

| **aaST number** | **ntST number** |
| --- | --- |
| **aaST01** | ntST01 ntST04 ntST05 ntST07 ntST08 ntST09 ntST10 ntST14 ntST17 ntST19 ntST20 ntST21 ntST22 ntST23 ntST25 ntST27 ntST36 ntST39 ntST47 ntST50 ntST51 ntST53 ntST55 ntST56 ntST59 |
| **aaST02** | ntST02 |
| **aaST03** | ntST03 |
| **aaST04** | ntST06 |
| **aaST05** | ntST11 |
| **aaST06** | ntST12 ntST15 ntST24 ntST26 ntST28 ntST37 ntST40 ntST41 ntST43 ntST45 ntST46 ntST49 ntST58 ntST60 ntST61 ntST62 ntST63 |
| **aaST07** | ntST13 ntST16 ntST35 ntST42 ntST54 |
| **aaST08** | ntST18 |
| **aaST09** | ntST29 |
| **aaST10** | ntST30 |
| **aaST11** | ntST31 ntST34 |
| **aaST12** | ntST32 |
| **aaST13** | ntST33 |
| **aaST14** | ntST38 ntST67 |
| **aaST15** | ntST44 |
| **aaST16** | ntST48 |
| **aaST17** | ntST52 |
| **aaST18** | ntST57 |
| **aaST19** | ntST64 |
| **aaST20** | ntST65 |
| **aaST21** | ntST66 |

CPV-2 antigenic variants and amino acid sequence types (aaSTs) obtained in this study.

| **Antigenic variant** | **aaST number** |
| --- | --- |
| **CPV-2a** | aaST01 aaST05 aaST07 aaST08 aaST09 aaST10 aaST11 aaST12 aaST16 aaST17 aaST18 aaST19 |
| **CPV-2b** | aaST02 aaST03 aaST04 aaST14 aaST21 |
| **CPV-2c** | aaST06 aaST13 aaST15 aaST20 |

CPV-2 antigenic variants, amino acid sequence types (aaSTs), nucleotide sequence types (ntSTs) and nucleotide sequences obtained in this study.

Sequences are named with: year of identification, lab ID number, antigenic variant and GenBank (https://www.ncbi.nlm.nih.gov/genbank/) accession number.

| **Antigenic variant** | **aaST number** | **ntST number** | **CPV-2 nucleotide sequences** |
| --- | --- | --- | --- |
| **CPV-2a** | **aaST01** | **ntST01** | 1994_0584_CPV-2a_AF306446 1995_0589_CPV-2a_MK348065 |
|  |  | **ntST04** | 1995_0618_CPV-2a_AF306447 1997_0660_CPV-2a_MK348066 1998_0679_CPV-2a_MK348067 1999_0687_CPV-2a_MK348068 2000_0697_CPV-2a_MK348069 2009_0212_CPV-2a_MK348076 2009_0272_CPV-2a_MK348077 2014_0577_CPV-2a_MK348093 |
|  |  | **ntST05** | 1996_0632_CPV-2a_AF306445 |
|  |  | **ntST07** | 1998_0677_CPV-2a_AF306448 |
|  |  | **ntST08** | 1999_0684_CPV-2a_KF373570 |
|  |  | **ntST09** | 1999_0685_CPV-2a_KF373571 |
|  |  | **ntST10** | 1999_0689_CPV-2a_KF373572 |
|  |  | **ntST14** | 2000_0701_CPV-2a_KF373574 2000_0704_CPV-2a_MK348070 2000_0706_CPV-2a_MK348071 |
|  |  | **ntST17** | 2001_0714_CPV-2a_KF373577 |
|  |  | **ntST19** | 2002_0312_CPV-2a_KF373579 |
|  |  | **ntST20** | 2003_0581_CPV-2a_KF373580 2009_0314_CPV-2a_MK348078 |
|  |  | **ntST21** | 2005_1805_CPV-2a_KF373581 |
|  |  | **ntST22** | 2006_0258_CPV-2a_KF373582 |
|  |  | **ntST23** | 2006_0691_CPV-2a_KF373583 |
|  |  | **ntST25** | 2007_0236_CPV-2a_KF373585 2008_0059_CPV-2a_MK348075 |
|  |  | **ntST27** | 2007_0733_CPV-2a_KF373587 2007_0996_CPV-2a_MK348074 |
|  |  | **ntST36** | 2009_0109_CPV-2a_KF373596 |
|  |  | **ntST39** | 2009_X8309_CPV-2a_KF385390 |
|  |  | **ntST47** | 2009_X60666_CPV-2a_KF385387 |
|  |  | **ntST50** | 2010_0409_CPV-2a_KF373611 |
|  |  | **ntST51** | 2010_0540_CPV-2a_KF373610 |
|  |  | **ntST53** | 2011_0042_CPV-2a_KF373600 2011_0055_CPV-2a_MK348085 |
|  |  | **ntST55** | 2011_0088_CPV-2a_KF373601 |
|  |  | **ntST56** | 2012_0149_CPV-2a_KF373606 |
|  |  | **ntST59** | 2012_0304_CPV-2a_KF373607 |
|  | **aaST05** | **ntST11** | 2000_0690_CPV-2a_KF373573 |
|  | **aaST07** | **ntST13** | 2000_0699_CPV-2a_AF393506 |
|  |  | **ntST16** | 2001_0709_CPV-2a_KF373576 2001_0710_CPV-2a_MK348072 |
|  |  | **ntST35** | 2008_0947_CPV-2a_KF373595 |
|  |  | **ntST42** | 2009_X29451_CPV-2a_KF385391 |
|  |  | **ntST54** | 2011_0053_CPV-2a_KF373605 |
|  | **aaST08** | **ntST18** | 2002_0305_CPV-2a_KF373578 |
|  | **aaST09** | **ntST29** | 2008_0060_CPV-2a_KF373589 |
|  | **aaST10** | **ntST30** | 2008_0096_CPV-2a_KF373590 |
|  | **aaST11** | **ntST31** | 2008_0097_CPV-2a_KF373591 |
|  |  | **ntST34** | 2008_0332_CPV-2a_KF373594 |
|  | **aaST12** | **ntST32** | 2008_0329_CPV-2a_KF373592 |
|  | **aaST16** | **ntST48** | 2009_X83090_CPV-2a_KF385388 |
|  | **aaST17** | **ntST52** | 2010_X987_CPV-2a_KF385389 |
|  | **aaST18** | **ntST57** | 2012_0151_CPV-2a_KF373603 |
|  | **aaST19** | **ntST64** | 2014_0791_CPV-2a_MK348096 |
| **CPV-2b** | **aaST02** | **ntST02** | 1995_0598_CPV-2b_KF373568 |
|  | **aaST03** | **ntST03** | 1995_0603_CPV-2b_KF373569 1995_0616_CPV-2b_AF306449 |
|  | **aaST04** | **ntST06** | 1996_0637_CPV-2b_AF306450 |
|  | **aaST14** | **ntST38** | 2009_0461_CPV-2b_KF373599 2009_0462_CPV-2b_MK348080 2009_0463_CPV-2b_MK348081 2010_0115_CPV-2b_MK348082 2010_0120_CPV-2b_MK348083 2010_0121_CPV-2b_MK348084 2013_0392_CPV-2b_MK348090 2015_1151_CPV-2b_MK348097 2016_1251_CPV-2b_MK348100 2016_1305_CPV-2b_MK348101 2017_0934_CPV-2b_MK348106 |
|  |  | **ntST67** | 2017_0850_CPV-2b_MK348104 2017_0851_CPV-2b_MK348105 |
|  | **aaST21** | **ntST66** | 2016_1418_CPV-2b_MK348102 |
| **CPV-2c** | **aaST06** | **ntST12** | 2000_0695_CPV-2c_AF401519 2006_0586_CPV-2c_MK348073 2017_0847_CPV-2c_MK348103 |
|  |  | **ntST15** | 2001_0707_CPV-2c_KF373575 |
|  |  | **ntST24** | 2006_1401_CPV-2c_KF373584 |
|  |  | **ntST26** | 2007_0241_CPV-2c_KF373586 |
|  |  | **ntST28** | 2007_1447_CPV-2c_KF373588 |
|  |  | **ntST37** | 2009_0326_CPV-2c_MK348079 2009_0800_CPV-2c_KF373597 |
|  |  | **ntST40** | 2009_X9896_CPV-2c_MK348107 2009_X23782_CPV-2c_MK348108 2009_X60282_CPV-2c_MK348113 2009_X60289_CPV-2c_MK348114 2009_X60298_CPV-2c_MK348115 2009_X60301_CPV-2c_MK348116 2009_X60304_CPV-2c_MK348117 2009_X60318_CPV-2c_MK348118 2009_X60384_CPV-2c_MK348121 2009_X78380_CPV-2c_KF385383 2009_X78391_CPV-2c_MK348125 |
|  |  | **ntST41** | 2009_X25835_CPV-2c_KF385385 2009_X25965_CPV-2c_MK348109 2009_X38718_CPV-2c_MK348110 |
|  |  | **ntST43** | 2009_X45361_CPV-2c_MK348111 2009_X60327_CPV-2c_MK348119 2009_X60339_CPV-2c_MK348120 2009_X60414_CPV-2c_MK348122 2009_X78184_CPV-2c_MK348123 2012_0134_CPV-2c_KF373602 2012_0138_CPV-2c_MK348086 |
|  |  | **ntST45** | 2009_X60406_CPV-2c_KF385384 |
|  |  | **ntST46** | 2009_X60416_CPV-2c_KF385386 2009_X78375_CPV-2c_MK348124 |
|  |  | **ntST49** | 2010_0118_CPV-2c_KF373598 2012_0263_CPV-2c_MK348087 2013_0570_CPV-2c_MK348092 2014_0608_CPV-2c_MK348094 |
|  |  | **ntST58** | 2012_0294_CPV-2c_KF373604 |
|  |  | **ntST60** | 2012_0334_CPV-2c_KF373608 2012_0340_CPV-2c_MK348088 2012_0341_CPV-2c_MK348089 |
|  |  | **ntST61** | 2013_0397_CPV-2c_KF373609 |
|  |  | **ntST62** | 2013_0538_CPV-2c_MK348091 |
|  |  | **ntST63** | 2014_0673_CPV-2c_MK348095 |
|  | **aaST13** | **ntST33** | 2008_0331_CPV-2c_KF373593 |
|  | **aaST15** | **ntST44** | 2009_X60203_CPV-2c_KF385382 2009_X60230_CPV-2c_MK348112 |
|  | **aaST20** | **ntST65** | 2015_1154_CPV-2c_MK348098 2016_1203_CPV-2c_MK348099 |
