# Supplementary material for: Molecular epidemiology of canine parvovirus type 2 in Italy from 1994 to 2017: recurrence of the CPV-2b variant
Source: BMC Vet Res. 2019 Nov 4;15:393. doi: 10.1186/s12917-019-2096-1 (PMC6829998; doi:10.1186/s12917-019-2096-1)
Supplement: Supplementary file 2 — Additional file 2 Sequence variability and Simpson’s index of the canine parvovirus type 2c (CPV-2c) sequences without Sicilian strains and of the CPV-2c Sicilian sequences. a: total number of non-synonymous differences. a/η: total number of non-synonymous differences on the total number of mutations. CPV-2: canine parvovirus type 2. D: Simpson’s index. k: average number of nucleotide differences. η: total number of mutations. π: nucleotide diversity (average number of nucleotide differences per site) and standard deviation. s: total number of synonymous differences. S: number of polymorphic (segregating) sites. [file 12917_2019_2096_MOESM2_ESM.docx]

Sequence variability and Simpson’s index of the CPV-2c sequences without Sicilian strains and of the CPV-2c Sicilian sequences.

|  | **No. of sequences** | **Total no. of sites** | ***S*** | **η** | **π** | **s** | **a** | **a/η** | **k** | **D** |
| --- | --- | --- | --- | --- | --- | --- | --- | --- | --- | --- |
| **CPV-2c without Sicilian sequences** | 25 | 1745 | 21 | 21 | 0.00170 SD 0.00018 | 19 | 2 | 0.09524 | 2.960 | 0.05 |
| **Sicilian CPV-2c** | 24 | 1745 | 5 | 5 | 0.00069 SD 0.00010 | 4 | 1 | 0.2 | 1.210 | 0.25362 |

*S*: number of polymorphic (segregating) sites;

η: total number of mutations

π: nucleotide diversity (average number of nucleotide differences per site) and standard deviation

s: total number of synonymous differences

a: total number of non-synonymous differences

a/η: total number of non-synonymous differences on the total number of mutations

k: average number of nucleotide differences

D: Simpson's index
