# Supplementary figures and images for: Molecular epidemiology of canine parvovirus type 2 in Italy from 1994 to 2017: recurrence of the CPV-2b variant
Source: BMC Vet Res. 2019 Nov 4;15:393. doi: 10.1186/s12917-019-2096-1 (PMC6829998; doi:10.1186/s12917-019-2096-1)

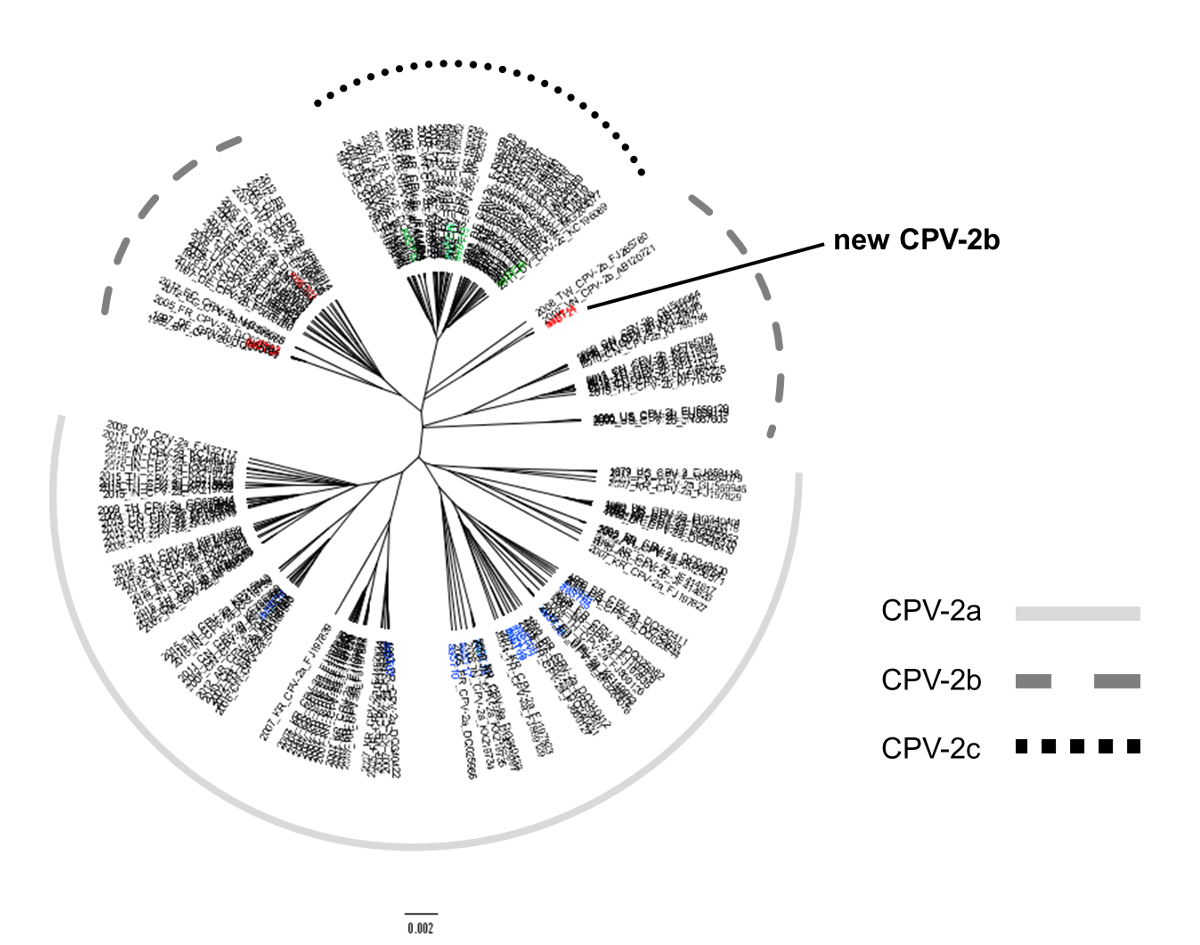

Supplement: Supplementary file 3 — Additional file 3. The unrooted phylogenetic tree constructed on VP2 amino acid sequence types obtained in this study and worldwide non-Italian canine parvovirus type 2 (CPV-2) amino acid reference sequences. A coalescent constant population tree was estimated using BEAST 1.10.4 and BEAGLE 3.1.0 software utilising a strict clock model with a coalescent constant population. The Jones-Taylor-Thornton (JTT) model with gamma distribution was used to build the phylogenetic trees. Phylogenetic analysis was carried out using the “strict clock” as a clock model; 175 worldwide non-Italian CPV-2 amino acid reference sequences (Additional file 4) were included in the analysis. The Bayesian Markov chain Monte Carlo (MCMC) chain lengths were 40,000,000 generations, with sampling every 2000 generations. The tree iteration was discharged with 10% of the chains as a burn-in pattern by using a tree annotator and the resulting MCMC tree was drawn using FigTree software 1.4.2. In blue, the CPV-2a aaSTs obtained in this study. In red, the CPV-2b aaSTs obtained in this study. In green the CPV-2c aaSTs obtained in this study. [file 12917_2019_2096_MOESM3_ESM.tif]
