# Supplementary material for: Molecular epidemiology of canine parvovirus type 2 in Italy from 1994 to 2017: recurrence of the CPV-2b variant
Source: BMC Vet Res. 2019 Nov 4;15:393. doi: 10.1186/s12917-019-2096-1 (PMC6829998; doi:10.1186/s12917-019-2096-1)
Supplement: Supplementary file 4 — Additional file 4. List of the 175 worldwide non-Italian canine parvovirus type 2 (CPV-2) reference sequences retrieved from the GenBank database and used for sequence analysis. Sequences are reported with: year of identification, acronym of the nation, antigenic variant and GenBank accession number. [file 12917_2019_2096_MOESM4_ESM.docx]

List of the 175 worldwide non-Italian CPV-2 reference sequences retrieved from the GenBank database (https://www.ncbi.nlm.nih.gov/genbank/) and used for sequence analysis.

Sequences are named with: year of identification, acronym of nation, antigenic variant and GenBank accession number.

2008_AR_CPV-2c_JF414819

2003_AR_CPV-2b_JF414817

2003_AR_CPV-2a_JF346754

2012_AR_CPV-2c_KM236568

2008_AR_CPV-2c_JF414818

2009_AR_CPV-2c_JF414821

2010_AR_CPV-2c_JF414826

2009_AR_CPV-2c_JF414820

2012_AR_CPV-2a_KM236573

2012_AR_CPV-2a_KM236571

2013_AR_CPV-2c_KM236569

2012_AR_CPV-2a_KM236570

2010_AR_CPV-2c_JF414825

2010_AR_CPV-2c_JF414824

2000_BR_CPV-2a_DQ340433

1995_BR_CPV-2a_DQ340431

1995_BR_CPV-2a_DQ340430

1993_BR_CPV-2a_DQ340422

1990_BR_CPV-2a_DQ340412

1990_BR_CPV-2a_DQ340411

1986_BR_CPV-2a_DQ340410

1985_BR_CPV-2b_DQ340409

1980_BR_CPV-2a_DQ340404

2002_CN_CPV-2b_GU569944

2013_CN_CPV-2a_KJ674820

2007_CN_CPV-2a_EU213085

2001_CN_CPV-2a_GU569946

2008_CN_CPV-2a_FJ432717

2013_CN_CPV-2a_KJ674814

2009_CN_CPV-2c_GU380305

2006_CN_CPV-2a_GQ169540

2015_CN_CPV-2b_MF467233

2015_CN_CPV-2a_MF467226

2015_CN_CPV-2c_MF467225

2010_CN_CPV-2b_KF785798

2012_CN_CPV-2a_KF785796

2010_CN_CPV-2a_KF785792

2011_CN_CPV-2b_KF785791

2011_CN_CPV-2a_KF785790

2010_CN_CPV-2a_KF785789

2010_CN_CPV-2b_KF785788

1997_DE_CPV-2c_FJ005196

1998_DE_CPV-2c_FJ005203

1997_DE_CPV-2b_FJ005261

1997_DE_CPV-2c_FJ005199

1997_DE_CPV-2b_FJ005260

2012_EC_CPV-2b_KF149985

2012_EC_CPV-2c_KF149984

2012_EC_CPV-2a_KF149978

2012_EC_CPV-2c_KF149962

2012_EC_CPV-2b_KF149972

2012_EC_CPV-2a_KF149973

2012_EC_CPV-2c_KF149966

2017_EC_CPV-2_MG264079

2017_EC_CPV-2b_MG264078

2017_EC_CPV-2c_MG264077

2017_EC_CPV-2a_MG264076

2006_ES_CPV-2c_FJ005214

2012_ES_CPV-2c_KP682513

2013_ES_CPV-2b_KP682514

2013_ES_CPV-2c_KP682516

2012_ES_CPV-2b_KP682512

2011_ES_CPV-2c_KP682511

2008_ES_CPV-2c_FJ005246

2005_FR_CPV-2a_DQ025997

2005_FR_CPV-2b_DQ025992

2005_FR_CPV-2a_DQ025986

2005_FR_CPV-2a_DQ025982

2005_FR_CPV-2b_DQ025961

2005_FR_CPV-2c_DQ025954

2005_FR_CPV-2a_DQ025952

2005_FR_CPV-2a_DQ025944

2005_FR_CPV-2a_DQ025943

2005_FR_CPV-2c_DQ025942

2008_GR_CPV-2c_GQ865518

2013_ID_CPV-2c_LC216904

2016_IN_CPV-2a_KX469435

2016_IN_CPV-2a_KX469434

2011_IN_CPV-2b_KX469432

2015_IN_CPV-2a_KX425922

2010_IN_CPV-2b_KX425921

2010_IN_CPV-2c_KX425920

2015_IN_CPV-2a_KX219742

2015_IN_CPV-2a_KX219741

2015_IN_CPV-2a_KX219740

2015_IN_CPV-2a_KX219739

2012_IN_CPV-2a_KX219736

2006_IN_CPV-2a_KX219735

2006_IN_CPV-2a_KX219734

2015_IN_CPV-2a_KX219733

2015_IN_CPV-2a_KX219732

2007_KR_CPV-2a_FJ197845

2007_KR_CPV-2a_FJ197844

2007_KR_CPV-2a_FJ197842

2007_KR_CPV-2a_FJ197841

2007_KR_CPV-2a_FJ197840

2007_KR_CPV-2a_FJ197839

2007_KR_CPV-2a_FJ197838

2007_KR_CPV-2a_FJ197837

2007_KR_CPV-2a_FJ197836

2007_KR_CPV-2a_FJ197835

2007_KR_CPV-2a_FJ197834

2007_KR_CPV-2a_FJ197833

2007_KR_CPV-2a_FJ197832

2007_KR_CPV-2a_FJ197831

2007_KR_CPV-2a_FJ197830

2007_KR_CPV-2a_FJ197829

2007_KR_CPV-2a_FJ197828

2007_KR_CPV-2a_FJ197827

2007_KR_CPV-2a_FJ197826

2007_KR_CPV-2a_FJ197825

2007_KR_CPV-2a_FJ197824

2007_KR_CPV-2a_FJ197823

2014_KR_CPV-2b_KP893078

2007_KR_CPV-2b_KP893077

2012_PT_CPV-2c_KR559893

2012_PT_CPV-2a_KR559891

2008_TH_CPV-2a_GQ379049

2009_TH_CPV-2a_GQ379048

2008_TH_CPV-2a_FJ869126

2009_TH_CPV-2a_GQ379044

2003_TH_CPV-2b_FJ869135

2004_TH_CPV-2a_FJ869129

2004_TH_CPV-2a_FJ869127

2008_TH_CPV-2b_FJ869122

2015_TH_CPV-2b_KP715715

2015_TH_CPV-2b_KP715712

2015_TH_CPV-2b_KP715706

2015_TH_CPV-2b_KP715695

2015_TH_CPV-2b_KP715694

2015_TH_CPV-2b_KP715693

2015_TH_CPV-2b_KP715687

2015_TH_CPV-2a_KP715681

2015_TH_CPV-2a_KP715671

2015_TH_CPV-2a_KP715670

2015_TH_CPV-2a_KP715669

2015_TH_CPV-2a_KP715666

2015_TH_CPV-2a_KP715663

2015_TH_CPV-2a_KP715660

2015_TH_CPV-2a_KP715659

2005_TW_CPV-2b_AY869724

2008_TW_CPV-2b_FJ265780

2007_TW_CPV-2b_EF592511

2007_US_CPV-2c_FJ005235

2009_US_CPV-2b_JN867603

1993_US_CPV-2a_M24000

2003_US_CPV-2a_AY742953

2008_US_CPV-2b_JN867607

2008_US_CPV-2b_JN867604

2009_US_CPV-2b_JN867605

2007_US_CPV-2c_FJ005236

2009_US_CPV-2b_JN867606

2008_US_CPV-2b_JN867602

1998_US_CPV-2b_EU659121

1998_US_CPV-2b_EU659120

2000_US_CPV-2b_EU659119

1981_US_CPV-2a_EU659118

1979_US_CPV-2_EU659116

2011_UY_CPV-2a_KC196114

2011_UY_CPV-2a_KC196110

2008_UY_CPV-2c_KC196105

2006_UY_CPV-2c_KC196099

2011_UY_CPV-2c_KC196091

2011_UY_CPV-2c_KC196090

2011_UY_CPV-2c_KC196089

2006_UY_CPV-2c_KC196087

2006_UY_CPV-2c_KC196086

2007_UY_CPV-2c_KC196082

2007_UY_CPV-2c_KC196081

2007_UY_CPV-2c_KC196080

2009_UY_CPV-2c_KC196079

2002_VN_CPV-2c_AB120727

2002_VN_CPV-2b_AB120722

2002_VN_CPV-2b_AB120721

2002_VN_CPV-2b_AB120720
